# Highly sensitive volumetric single-molecule imaging

**DOI:** 10.1515/nanoph-2024-0152

**Published:** 2024-07-12

**Authors:** Le-Mei Wang, Jiah Kim, Kyu Young Han

**Affiliations:** CREOL, The College of Optics and Photonics, 6243 University of Central Florida, Orlando, FL, USA; Department of Cell and Developmental Biology, University of Illinois at Urbana-Champaign, Urbana, IL, USA

**Keywords:** volumetric imaging, single-molecules, single-particle tracking, extended depth-of-field, super-resolution imaging, PSF engineering

## Abstract

Volumetric subcellular imaging has long been essential for studying structures and dynamics in cells and tissues. However, due to limited imaging speed and depth of field, it has been challenging to perform live-cell imaging and single-particle tracking. Here we report a 2.5D fluorescence microscopy combined with highly inclined illumination beams, which significantly reduce not only the image acquisition time but also the out-of-focus background by ∼2-fold compared to epi-illumination. Instead of sequential *z*-scanning, our method projects a certain depth of volumetric information onto a 2D plane in a single shot using multi-layered glass for incoherent wavefront splitting, enabling high photon detection efficiency. We apply our method to multi-color immunofluorescence imaging and volumetric super-resolution imaging, covering ∼3–4 µm thickness of samples without *z*-scanning. Additionally, we demonstrate that our approach can substantially extend the observation time of single-particle tracking in living cells.

## 1 Introduction

Fast volumetric imaging is essential for biological studies since it not only preserves high spatial resolution but also conserves information in the time domain, enabling one to investigate cell dynamics and subcellular structures effectively [1]. Nevertheless, the image acquisition speed is often limited by the speed of a bulky scanning piezo stage along the *z* direction [2]. To overcome this dilemma, various strategies have been proposed. For example, the axial scanning speed was enhanced by remote focusing [3], tunable lens [4], [5], deformable mirror [6], or other fast focus devices [7]. Conversion of lateral scanning to axial scanning [8] or multi-angle projection [9] also significantly improved the volumetric imaging speed. However, these approaches inherently exhibit a relatively short detection duty cycle, namely, to obtain sufficient fluorescence signals an intense illumination intensity is required. Other approaches include multifocal imaging, which records images at different imaging planes simultaneously [10], or light-field microscopy, which captures spatial and angular information to reconstruct 3D images [11], [12]. However, they suffer from either a low signal-to-noise ratio (SNR) and/or degraded spatial resolution [2]. For instance, multiplane imaging with diffractive grating showed only 5 % detection efficiency compared to the single plane imaging [13].

Extended depth of field imaging through point spread function (PSF) engineering can address the aforementioned issues by fully leveraging the detection duty cycle. Recently, a phase pattern called a 2.5D phase mask on a spatial light modulator (SLM) demonstrated a highly uniform axial profile over an imaging depth of ∼5 µm with minimal broadening of lateral resolution and negligible side lobes [14]. Its enhanced detection efficiency and the capability of aberration correction make it suitable for quantitative high-resolution volumetric imaging [15]. Due to its reduced light dose, it decreased photobleaching [14], indicating that it can allow long-term live-cell imaging with minimal photodamage [16], [17]. Alternatively, using multiple annular glasses with a certain diameter and thickness successfully extended the imaging depth by five times [18], [19]. This was achieved by destroying the coherence of the fluorescent emission signal, thus creating Bessel-like beams with suppressed sidelobes. This method, known as the ‘layer cake,’ offers advantages in terms of easy installation and high transmission efficiency. Both approaches, hereinafter 2.5D microscopy (2.5DM) yielded promising results in single-shot RNA fluorescence *in situ* hybridization, fast high-throughput immunofluorescence imaging [14], and long-term live-cell imaging of microtubules [18].

However, 2.5DM truly demonstrates its merit when used in a relatively sparse condition because it projects all fluorescent signals, including unwanted background from the extended detection volume onto the 2D plane. Deconvolution [18] or deep-learning-based image reconstruction [20], [21] can enhance signal-to-background ratio (SBR), but they are likely to be error-prone for low fluorescent samples such as single-molecules. As of now, 2.5DM has not been experimentally explored in single-molecule localization microscopy (SMLM) [22], [23] and single-particle tracking (SPT) in live cells [24], [25], although theoretical [26], [27] and *in vitro* studies [28] have been conducted.

Here, we present a highly sensitive and high-resolution volumetric imaging technique that combines a highly inclined and laminated optical sheet (HILO) [29], [30] for excitation with a layer cake in the detection path. Unlike the previous studies where the entire volume was excited by epi-illumination, HILO illumination significantly reduces out-of-focus background, increasing image contrast and enabling robust deconvolution. The property of incoherent wavefront splitting facilitates multicolor volumetric fluorescence imaging without requiring changes to the phase plate. Additionally, we demonstrate 2.5D super-resolution imaging and visualize fine details spanning up to ∼3–4 µm of depth-of-field (DOF) without the need for multiple *z*-scanning. Finally, we utilize our 2.5DM for single-particle tracking and demonstrate ∼3–4-fold extended observation time of single-particle tracks in live-cells, which is often limited by the short tracking length due to the limited DOF.

## 2 Results

### 2.1 2.5D imaging with reduced background by HILO illumination

**Figure 1:**
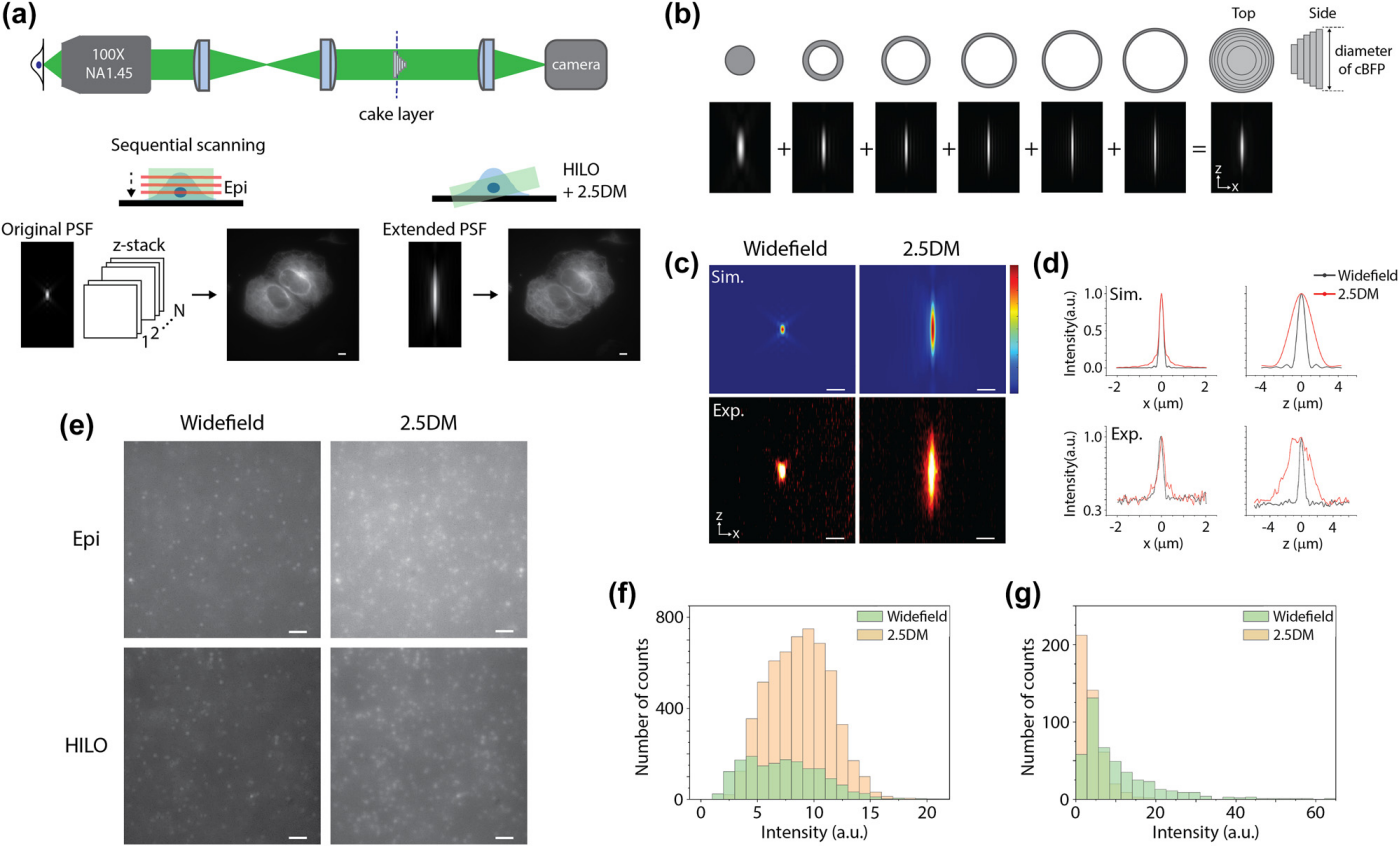
HILO illumination 2.5D imaging with a layer cake. (a) Comparison of epi-illumination widefield imaging and HILO illumination 2.5D extended depth of field microscopy. Projected images generated with multiple *z*-slices by widefield (left) and with 2.5DM (right). (b) Working principle of the layer cake. (c) Point spread functions at the *x*–*z* plane of widefield (left) and 2.5D detection (right) for simulated (top) and experimental (bottom) results with 100-nm red fluorescent beads. (d) Line profiles of (c) along the *x*-axis (left) and *z*-axis (right). (e) Fluorescence images of 100-nm red beads embedded in a 3D hydrogel captured with epi (top) and HILO illumination (bottom) using widefield (left) and 2.5DM (right). (f) Number of counted beads in the 3D hydrogel with 2.5DM or widefield from a single *z*-slice. (g) Comparison of the peak intensities for widefield and 2.5DM. Scale bars, 5 µm (a), 1 µm (c), and 2 µm (e).

We first measured the PSF of our optical imaging system where a five-layer cake was placed in the conjugate back focal plane of an objective lens (Figures 1(a) and S1). The layer cake is analogous to several annular apertures. Each layer is designed with a certain thickness to exceed the coherence length of the broadband fluorescence signal, allowing them to add up incoherently to generate an extended beam without interference (Figure 1(b)) [18]. For instance, with a single layer having a normalized diameter of ∼0.7 mounted on a glass substrate, both an annular beam and a central beam are produced with the equal intensity. While the central beam is elongated in the lateral and axial directions, resembling the effect of using a low numerical aperture lens, the annular beam results in an axially elongated but laterally sharper beam with sidelobes. When the two beams are combined, the effect of sidelobes is reduced, and the resulting PSF is elongated primarily in the axial direction. This approach allows for increasing the number of layers until the desired DOF and tolerable lateral resolution are achieved. We imaged 100-nm diameter red fluorescent beads (580/605) immobilized on a coverslip using 561 nm laser excitation. The full width at half maximum (FWHM) of lateral and axial profiles for 2.5DM were 395 nm and 3.55 µm while those for widefield were 275 nm and 686 nm, respectively (Figure 1(c)). It showed ∼5.2-times extension along the axial direction while there was a ∼1.4 times broadening in the lateral direction. Our measurements corresponded well to the simulation results which also showed ∼5 times extension along the *z* direction (Figure 1(d)).

Next, we prepared fluorescent beads in a 3D hydrogel and illuminated either an epi or HILO beam to measure their background levels. The illumination beam size was ∼40–50 µm, and the thickness of the HILO beam was ∼5–6 µm, which corresponds to an inclination angle of ∼80°. As reported before [14], the signal to background ratio (SBR) for 2.5DM was lower than for a single plane image of widefield because of its extended depth of field (Figure 1(e)). Surprisingly, HILO illumination demonstrated a lower background level compared to epi-illumination in 2.5DM (Figure 1(e), bottom). To quantify this difference, we calculated the SBR of multiple beads (*n* > 10) in the 2.5D images. The SBR for HILO was 2.1-fold higher than that for epi-illumination, indicating that our HILO illumination effectively reduces background without compromising imaging depth. The number of beads detected with 2.5DM was 4.4 times larger than that from a single *z*-slice using widefield detection (Figure 1(f)). We also measured the peak intensities of fluorescent beads for widefield and 2.5DM under the same exposure time and excitation intensity. As shown in Figure 1(g), the peak intensity of 2.5DM was 2.6 times lower than that of widefield, mainly due to the laterally broadened profile. This effect was also observed in our previous study using a 2.5D phase mask [14]. We can compensate for this loss by increasing either the exposure time or the illumination intensity for 2.5DM.

### 2.2 Multicolor immunofluorescence 2.5D imaging

A phase plate is typically designed for a monochromatic or narrow-band wavelength, making it impracticable for use in multicolor fluorescence imaging unless multiple phase plates are used for each fluorophore. In contrast, the layer cake is expected to be less affected by wavelength because it relies on the incoherent superposition of detected fluorescence. To ensure this, we measured the PSFs of yellow-green (*λ*_em_ = 515 nm), red (605 nm) and crimson (645 nm) beads. As shown in Figure S2, their axial profiles extended from 0.64 µm, 0.69 µm and 0.74 µm to 2.96 µm, 3.55 µm, and 4.03 µm, respectively. Overall, the layer cake showed ∼5.1 times extension of the depth of field with the minimal variation in shape. At longer wavelengths, the depth of field for 2.5DM increases similarly to widefield microscopy.

**Figure 2:**
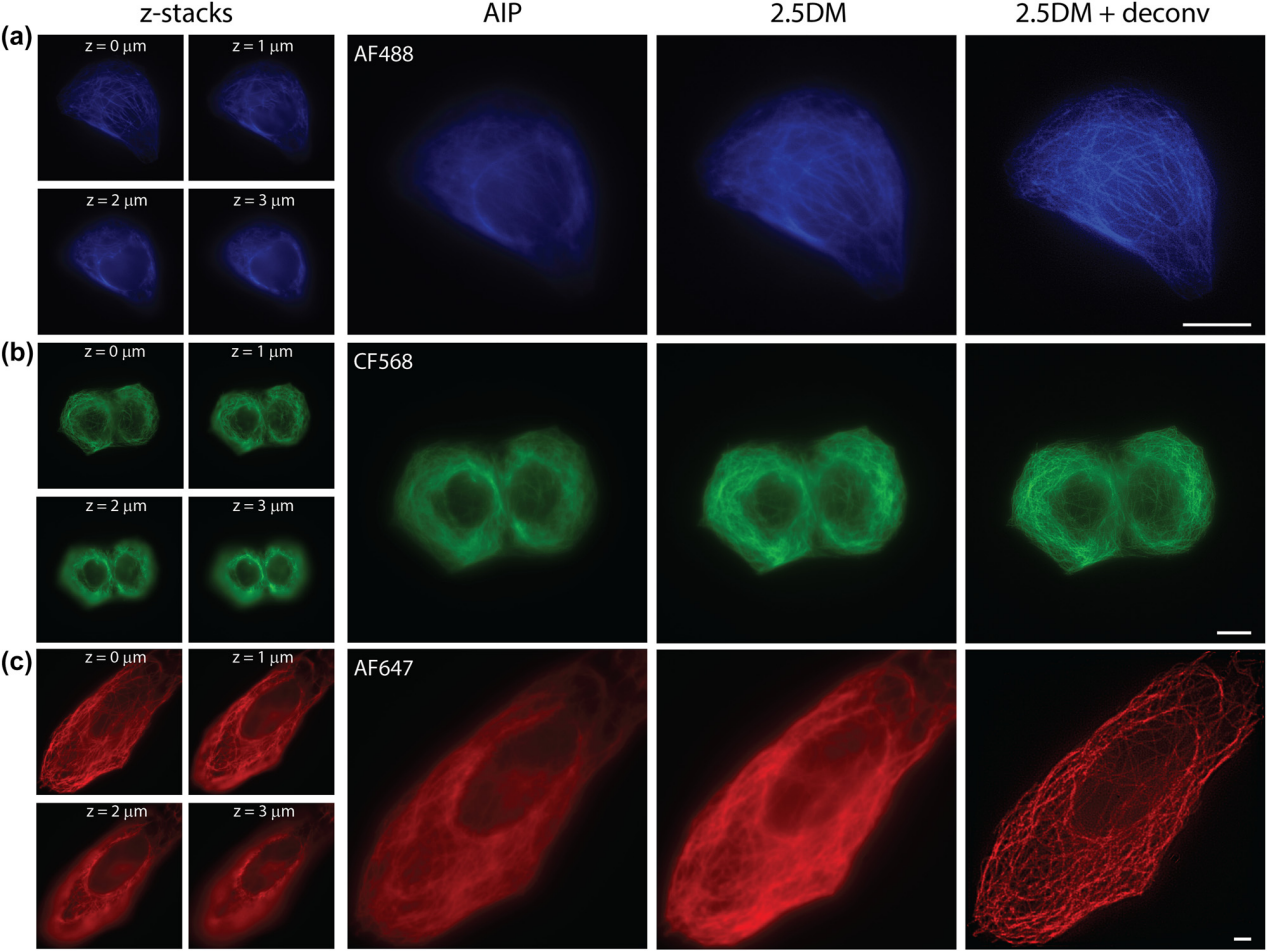
Multicolor 2.5D imaging with a layer cake. Immunofluorescence images of microtubules labeled with AF488 (a), CF568 (b), and AF647 (c) in U2OS cells captured at different depths, their average intensity projection (left), 2.5D images and their deconvoluted images (right). Scale bars, 10 µm.

We applied our 2.5DM to immunofluorescence imaging to visualize microtubules labeled with AF488, CF568 or AF647 in U2OS cells. For reference, we recorded 25 images at different *z* depths with a step size of 0.2 µm, covering a thickness of 5 µm using HILO illumination without the layer cake (Figure 2). The exposure time of each frame was set to 50 ms. The *z*-stack images were then projected onto a 2D image using average intensity projection (AIP). In comparison, a single shot 2.5D image was captured with 150 ms exposure time. As depicted in Figure 2, the 2.5D images for all three colors yielded comparable results to the AIP images of widefield in terms of structural details across the cell volume (∼5 µm). Notably, the image acquisition time was significantly reduced from ∼1,500 ms to 150 ms, despite the exposure time of 2.5DM being three times longer than widefield. It is inevitable for 2.5D images to have higher background; however, 2D deconvolution was able to substantially improve the contrast of the 2.5D images (Figure 2, right).

### 2.3 Volumetric SMLM imaging with 2.5DM

For whole-cell super-resolution imaging, single-molecule localization microscopy (SMLM) images are typically acquired at each *z*-plane with a step size of 0.3–0.5 µm over an imaging depth of 3–10 µm depending on the target molecules [31], [32]. To assess whether our 2.5D imaging approach can be used to obtain projected SMLM images without *z*-scanning, we imaged microtubule structures in U2OS cells with illumination of a 640 nm laser (*I* = 15 kW/cm^2^) and a sparse activation of a 405 nm laser. Three images at different *z* depths were captured without the layer cake at *z* = 0.5, 2, and 3.5 µm (Figure S3). With the layer cake added, a projected image was captured at *z* = 2 µm. Each stack contains 8,000 frames recorded with an exposure time of 10 ms.

**Figure 3:**
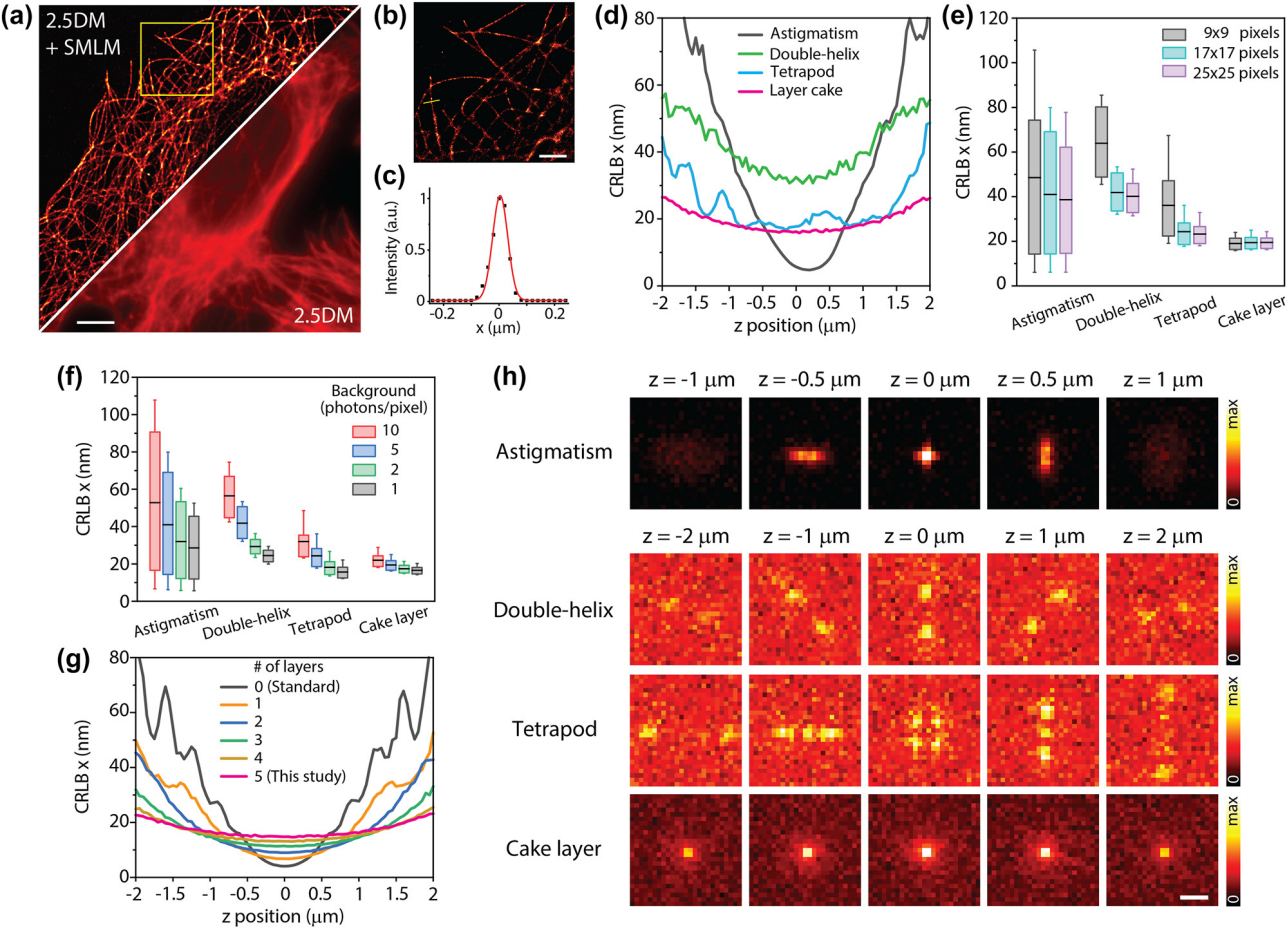
SMLM imaging with 2.5DM. Diffraction limited (a) and super-resolution (b) 2.5D immunofluorescence images of microtubules labeled with AF647 in U2OS cells. (b) A zoomed-in image from a subregion marked by a dashed square in (a). (c) An intensity profile from a solid line (yellow) in (b). (d–g) Cramér–Rao lower bound (CRLB) calculations for the estimation of the *x* coordinate of single-molecules. (d) CRLBs for astigmatism, double-helix, tetrapod, and layer cake at different *z* depths with *N* = 1,000 photons, *β* = 5 photons/pixel, 1 pixel = 160 nm, *λ* = 670 nm, numerical aperture = 1.4 and *ω* = 17 × 17 pixels. CRLBs calculated for different array sizes with *β* = 5 (e) and for various background levels (f). In boxplots, the black line represents the mean, and box edges represent upper and lower quartiles. The whisker extends between 10 and 90 percentiles. (g) CRLBs of layer cake for different number of layers. (h) Simulated PSFs used for CRLB calculations (*β* = 5). Five images were averaged. Scale bars, 10 µm (a), 2 µm (b), and 1 µm (f).

As depicted in Figure 3(a)–(c), the reconstructed SMLM image using 2.5DM showed significantly improved resolution compared to a regular 2.5DM image. Our result was comparable to the sum of three SMLM images (24,000 frames) obtained from serial *z*-scanning without the layer cake (Figure S3). The spatial resolution of structures was evaluated with line profile analysis, where conventional SMLM exhibited a resolution of 63 nm, while 2.5DM showed 77 nm (*n* = 10). A slightly lower resolution for 2.5DM is expected due to its broadened lateral resolution, resulting in a decrease in localization accuracy. It is worth noting that a *z*-drift correction module is unnecessary for 2.5DM.

To quantify the performance for estimating single-molecule localization precision, we calculated the Cramér–Rao lower bound (CRLB) [33] of our method and compared it with other 3D-SMLM approaches such as astigmatism [34], [35], double-helix [36], [37] and tetrapod [38], [39]. A few characteristics of the cake layer were noted. Firstly, the layer cake maintains a low CRLB over a large *z* range compared to other methods (Figure 3(d)). The standard deviation of the layer cake was the smallest among all the methods. Secondly, the layer cake is not strongly affected by the number of pixels (or the size of the array) due to the invariance of the PSF shape. This behavior is crucial for performing robust and precise localization at a reasonable density of single-molecules. For instance, as the array size decreases from 25 × 25 pixels (4 × 4 µm^2^) to 9 × 9 pixels (1.4 × 1.4 µm^2^), CRLBs for the layer cake exhibit 17.3 ± 2.5 nm and 17.4 ± 2.7 nm, respectively, whereas CRLBs for tetrapod changes from 22.3 ± 11.5 nm to 37.5 ± 34.7 nm (Figure 3(e)). Thirdly, the localization precision of the layer cake is resistant to elevated background noise compared to other methods (Figure 3(f)). This is likely due to the fact that the intensity of the layer cake is primarily distributed in the center region (Figure 3(h)). Importantly, for a fair comparison, we assumed that the number of detected photons for double-helix and tetrapod is 35 % of that for astigmatism and layer cake [15], [40] because the former typically use SLM, whereas the latter use passive optics with high transmission efficiencies.

### 2.4 Single-particle tracking with extended track lengths

**Figure 4:**
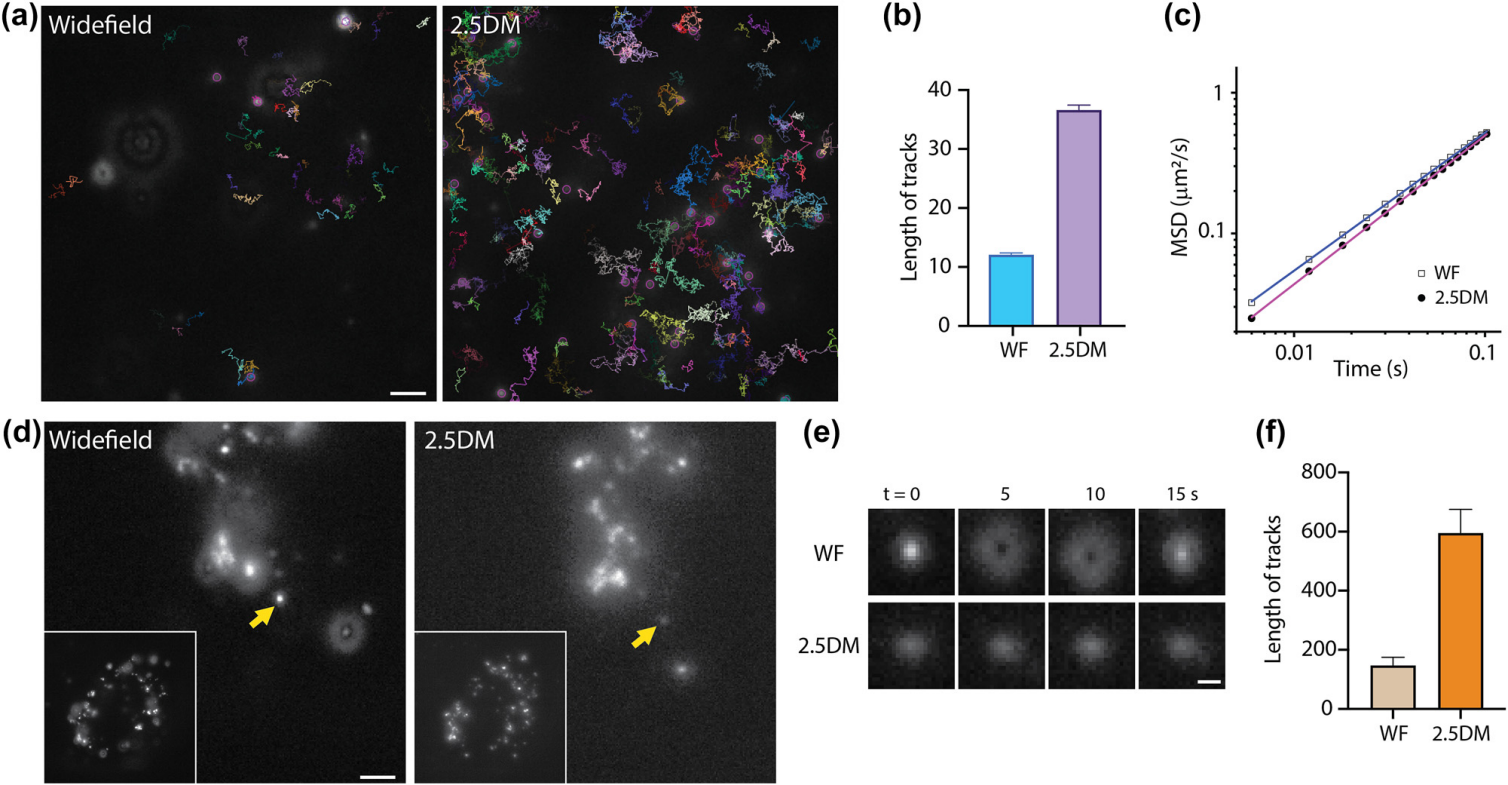
Extended observation in single-particle tracking by 2.5DM of beads with widefield microscopy and 2.5DM. (a) Representative images and trajectories of freely diffusing 100 nm beads in a 60 % TDE solution measured by widefield (left) or 2.5DM (right). Tracking lengths (b) and MSD curves (c) of beads measured with widefield or 2.5DM. (d) Fluorescence images of neutravidin coated 40-nm fluorescent beads in live U2OS cells. Inset: Whole cell images. (e) Time-lapse images of single-particles marked by arrows in (d). (f) The average tracking lengths for different particles (*n* = 10). Scale bars, 2 µm (a,d) and 0.5 µm (e).

Finally, we utilized our 2.5DM for single-particle tracking to assess whether it could prolong the observation time of tracks. We imaged 10,000 frames of 100-nm red fluorescent beads in a 60 % TDE solution under widefield or 2.5DM (Figure 4(a)) and analyzed the particle tracks. Two key observations emerged: (1) the number of beads detected with the layer cake increased by ∼4 times, and (2) the track length for 2.5DM was extended by ∼4.4 times (Figure 4(b) and Supplementary Movie 1). In contrast, for widefield, the number of particles detected was fewer, and the tracking length was shorter than with the layer cake, likely due to particles more easily traveling out of focus. Additionally, we analyzed the mean square displacement (MSD) of particles. The log–log plots for widefield and 2.5DM exhibited linear change over time [28], with slopes of 0.98 and 1.05, respectively (Figure 4(c)). Any slight discrepancy in their offsets is attributed to localization error [41].

**Supplementary Movie 1:** 

Next, we introduced neutravidin-coated fluorescent nanoparticles (40 nm) into live U2OS cells and observed their dynamics within the cytoplasm. Following endocytosis, they were observed within endosomes and/or lysosomes (Figure 4(d) and Supplementary Movie 2). Under widefield, only a few nanoparticles were in focus for a short period, making it challenging to track each particle (Figure 4(e)). In contrast, with 2.5DM, all nanoparticles appeared in-focus, and their fast dynamics were clearly visualized for ∼4-fold longer period (Figure 4(f)). Specifically, the durations of in-focus periods for widefield and 2.5DM were 147 and 595 frames, respectively. Thanks to its extended observation time, we were able to monitor diverse events. For instance, multiple nanoparticles were trapped, presumably in endosomes, but exhibited fast tumbling dynamics. Some of them showed trafficking between two regions, endocytosis dynamics and directional motion over a few micrometers.

**Supplementary Movie 2:** 

## 3 Discussion

In this study, we presented a 2.5D microscopy technique capable of readily achieving multicolor subcellular imaging, volumetric super-resolution imaging and single-particle tracking with extended track lengths. Unlike conventional widefield approaches that require scanning along the *z*-direction to collect volumetric information, 2.5DM provides comprehensive volume information in a single capture by projecting signals from various focal planes simultaneously. This highlights its capacity for fast, high-throughput image acquisition.

Unlike our previous works [14], [15], the layer cake cannot adaptively compensate for aberrations, and its axial profile is less uniform than that of a 2.5D phase mask. Its key merit lies in its high transmission efficiency (>95 %), whereas SLM-based approaches exhibit unavoidable losses of ∼59 % or ∼23 % in a single- or double-pass configuration, respectively. Nevertheless, both approaches showed similar localization precision (Figure S4). Unlike other passive phase plates, the layer cake offers some tunability. By using different focal length lens for L3 in Figure S1, the number of layers can be controlled, resulting in varying DOF and CRLB (Figure 4(g)). Surprisingly, since Abrahamsson and Gustafsson reported the use of layer cake [18], it has not been widely adopted in high-resolution volumetric imaging, possibly due to concerns about its high background. However, with HILO beam as the excitation source, we have demonstrated that the background level can be significantly reduced. This reduction in background also facilitates obtaining robust deconvolution results.

We adapted our work to single-molecule localization microscopy, and the image quality was comparable to conventional SMLM images obtained with maximum intensity projections (MIP) after collecting at multiple planes. Ideally, our approach can be employed to acquire volumetric SMLM imaging for any targets with a thickness of 4–5 µm in a short period of time. However, since our sample was incubated in a water-based medium during imaging, the effective projection depth was slightly reduced to ∼3–4 µm due to refractive index mismatch. This limitation can be alleviated by using a water immersion objective or a refractive index matched imaging buffer [42]. Since the projection covers a volume 4–5 times thicker than conventional imaging, the density of single molecules increases accordingly, posing challenges for accurate single-molecule localization, especially when emitters are highly overlapped. Implementing multi-emitter fitting algorithms [43] or deep-learning-based approaches [44] can help resolve localization issues arising from high density. Furthermore, it is possible to accelerate image acquisition by simply increasing the excitation laser intensity [45], which can be readily extended to high-throughput nanoscopy [46] with volumetric imaging.

The greatest advantage of our method is that we maintain focus during single-particle tracking for extended periods. SPT has long suffered from limited tracking length, as targets often move out of focus quickly, hindering robust analysis of biomolecule dynamics [47], [48]. Our results showed track lengths extended by at least four times, enabling powerful analysis of complex dynamics, such as those exhibited by fast-moving biomolecules [49] or vesicles [50]. Our approach may be applicable to single-particle tracking with scattering [51]. Intensity projection is commonly used to represent 3D information in bioimages [52], meaning that when the depth information is less crucial compared to overall structure, 2.5DM can be a more efficient strategy. Since the layer cake is commercially available and cost-effective, and HILO illumination is relatively easy to implement, we anticipate that our method will see wide adoption in single-molecule volumetric imaging as well as high-throughput, high-resolution multicolor volumetric imaging.

## 4 Materials and methods

### 4.1 Preparation of fluorescent bead samples

For the 2D bead samples, yellow-green, red or crimson beads (F8803, F8801, F8806; ThermoFisher) were immobilized on the poly-l-lysine (P8920; Sigma) coated coverslips. The coverslip was sealed with a glass slide after ProLong Diamond antifade mountant (P36961; ThermoFisher) was injected. The 2D bead samples were imaged after 24 h when the mounting medium was fully solidified. For the 3D hydrogel samples, a hydrogel was prepared as previously [53]. Briefly, we used 7.5 % acrylamide:bisacrylamide (29:1) (National Diagnostics), 0.2 % (v/v) tetramethylethylenediamine (TEMED; T7024, Sigma) and 0.02 % (w/v) ammonium persulfate (A3678; Sigma) in a 0.5× TAE buffer. Red fluorescent beads were added at a concentration of 5 % (v/v) to the final hydrogel solution. The solution was quickly injected to a flow-chamber after being fully mixed with a vortex mixer. Images of the 3D hydrogel samples were captured after 10 min of solidifying process. For tracking of freely diffusing single-particles, red fluorescent beads were sonicated and added to a 60 % TDE (2,2′-thiodiethanol; 166782; Sigma) in water. The mixed solution was injected into a flow chamber and imaged right after the injection. The refractive index of the final solution is ∼1.45 [54].

### 4.2 Cell culture and immunostaining

U2OS cells (HTB-96; ATCC) were grown at 37 °C with 5 % CO_2_ in a humidified incubator. The cells were cultured using McCoy’s 5A medium (SH30200; Cytiva), supplemented with 10 % fetal bovine serum (F0926, Sigma) and 1 % penicillin/streptomycin (15140122; ThermoFisher). Prior to fixation, the U2OS cells were subcultured in a petri-dish (P35G-1.5-14-C; MATTEK) for 24–48 h. For fixation, we first added 0.6 % paraformaldehyde solution (PFA, 15750; Electron Microscopy Sciences), 0.1 % glutaraldehyde (16019; Electron Microscopy Sciences), Triton X-100 (93443; Sigma), and 1× phosphate buffered saline (PBS), incubated under 37 °C for 1 min, and added 4 % PFA solution, 0.2 % glutaraldehyde, and 1× phosphate buffered saline (PBS) under room temperature for 15 min. Then the cells were incubated in 0.1 % sodium borohydride (213462; Sigma) at room temperature for 10 min. After washing three times with 1× PBS, the sample was incubated with the blocking solution made of BSA (37525; ThermoFisher) and Triton X-100 in 1× PBS for 1 h. Then the sample was incubated with mouse anti-β-tubulin (T5293, Sigma) at 4 °C overnight. After washing three times with 1× PBS, the sample was incubated with goat anti-mouse secondary antibodies labeled with Alexa Fluor 488 (AF488, A11001; ThermoFisher), CF568 (20800; Biotium) or Alexa Fluor 647 (AF647, A21235, ThermoFisher) for 2 h at room temperature. Post-fixation was done with 4 % PFA in 1× PBS for 15 min. After three washes, the samples were imaged in 1× PBS.

For SMLM imaging, U2OS cells immunostained with AF647-labeled secondary antibodies were imaged in an imaging buffer containing 1 % β-mercaptoethanol (BME, 63689, Sigma), 1 % oxygen scavenger (glucose oxidase, catalase and dextrose) in 50 mM Tris (pH 8) and 10 mM NaCl. For single-particle tracking in live cells, we plated U2OS cells in 35 mm dishes for 24 h before introducing 40 nm neutravidin-coated fluorescent beads (F8770, ThermoFisher). The bead solution was diluted 1:100 or 1:1,000 in culture medium and added to the plated cells. The cells initiated bead uptake within 1∼2 h through endocytosis [55]. To determine the optimized number of beads loaded within cells, we incubated them for 6–24 h in a cell incubator. Prior to imaging, we replaced the culture media with a fresh media without phenol red.

### 4.3 Optical setup

We used a custom-made microscope as shown in Figure S1 with modified excitation and detection paths for HILO illumination and 2.5D imaging. Lasers (*λ* = 488/561/638 nm; Cobolt) from a single mode fiber were collimated by a lens (L1, *f* = 60 mm) and the beam size was controlled through an iris. The excitation beam was focused by a lens (L2, *f* = 300 mm), reflected by a dichroic beamsplitter (TRF8991, Chroma), and focused to the back focal plane of the objective lens (UPlanXApo, 100×/1.45 Oil; Olympus). To generate an inclined beam and tune its angle, a mirror (M2) was placed at the conjugate imaging plane. For SMLM imaging, a red laser (*λ* = 640 nm; Cobolt Bolero) was adopted to provide high power illumination, and an activation beam (*λ* = 405 nm; Cobolt) whose illumination frequency was controlled by a function generator was sent to the sample. A flat top shaper (TSM25-10-S-B; Asphericon) was introduced to shape the excitation intensity uniformly across the field-of-view [56]. Fluorescence signals were captured through the same objective lens, passed through a multi-band pass filter, and imaged by a scientific CMOS camera (Zyla 4.2 PLUS; Andor) together with a tube lens (L4, *f* = 180 mm) and a relay imaging system (L5, *f* = 400 mm; L6, *f* = 600 mm) with a 4*f* configuration. The one-pixel size is ∼43 nm. We used the center part of the camera with 1,024 × 1,024 pixels and 512 × 512 pixels for SMLM imaging and single-particle tracking, respectively, while for other image acquisitions we used 2,048 × 2,048 pixels. The elongated PSF was implemented by installing a layer cake (OP-12301-400-700, Light Machinery) at the conjugate back focal plane of the objective between L5 and L6. The multi-layered glass is constructed from fused silica, featuring five layers on top of a base circular glass layer. Each layer has a different radius to ensure a similar amount of transmitted light, with diameters of 5.254, 7.276, 8.736, 9.874, and 10.803 mm, respectively. Each layer is 1.33 mm thickness, and the flat side has anti-reflective coating for visible wavelengths.

### 4.4 Simulation and imaging analysis

To predict and verify our imaging system, we simulated the detection PSF using Fourier optics by MATLAB [57], [58]. The intensities of bead samples were measured in 5 × 5 pixels surrounding the peak of each bead and calculated over 400 beads. Immunofluorescence 2.5DM images were deconvolved with Huygens Essential 23.10 (Scientific Volume Imaging B.V.) using classic MLE algorithm, with acuity: 40, SNR: 43.52, background value: 110, quality threshold: 0.2, and iterations: 15. SMLM images were reconstructed with SMAP software [59]. The images were reconstructed using Difference of Gaussian filter (DoG) with sigma 1.2 and *xy*-drift correction with 15 time points after the localization process.

For the Cramér–Rao lower bound (CRLB) calculation, we adopted the same method that previously used [33], [38], except that we employed a vectorial PSF model [14] assuming a freely rotating emitter. We used the following simulation parameters: *N* = 1,000 photons; Poissonian background (*β*) = 1, 2, 5 or 10 photons/pixel; numerical aperture of objective = 1.4; refractive index = 1.518; wavelength (*λ*) = 670 nm; pixel size = 160 nm; array size (*ω*) = 9 × 9, 17 × 17 or 25 × 25 pixels. In consideration of the use of SLM for double-helix and tetrapod, their number of photons was reduced to 35 %. The double-helix PSF was calculated by six rings of azimuthal ramps [37], and the tetrapod PSF was obtained by CRLB optimization over a 4 μm *z* range [38], [39].

### 4.5 Single-particle tracking analysis

We employed the TrackMate plugin [60] in Fiji software for our analysis. A sub-pixel localization and a Laplacian of Gaussian (LoG) detector were utilized with a particle size set to 5 pixels, a quality filter of 50, and a signal-to-noise ratio threshold of 0.75. For widefield samples, we applied an additional contrast filter by selecting particles with values above 0.75 to exclude out-of-focus ring patterns. Then we linked localized spots using the simple linear assignment problem (LAP) tracker with a maximum linking distance of 20 pixels. To analyze MSD, we used the u-track software [61] with default settings in the “single molecule tracking” method. We set the maximum gap to close between frames to zero. For MSD calculation, we analyzed tracks longer than four time points using a custom MATLAB script.

## Supplementary Material

Supplementary Material Details
