# Supplementary material for: Highly sensitive volumetric single-molecule imaging
Source: Nanophotonics. 2024 Jul 12;13(20):3805–14. doi: 10.1515/nanoph-2024-0152 (PMC11366074; doi:10.1515/nanoph-2024-0152)
Supplement: Supplementary file 5 — Supplementary Material Details [file j_nanoph-2024-0152_suppl_001.pdf]

Supplementary Information

Highly sensitive volumetric single-molecule imaging

Le-Mei Wang[1], Jiah Kim[2] and Kyu Young Han[1]*

[1]CREOL, The College of Optics and Photonics, University of Central Florida, Orlando, Florida, USA.

[2]Department of Cell and Developmental Biology, University of Illinois at Urbana-Champaign, Urbana, IL, USA

*Correspondence should be addressed to K.Y.H. (kyhan@creol.ucf.edu).

Supplementary Movies

Supplementary video 1. Time lapse images and trajectories of freely diffusing 100-nm beads in a 60% TDE solution measured at 91 frames per second widefield (left) and 2.5DM (right). Scale bar, 2 μm.

Supplementary video 2. Time lapse images of neutravidin coated 40-nm beads in live U2OS cells measured at 40 frames per second by widefield (left) and 2.5DM (right). Scale bar, 2 μm.

Supplementary Figures

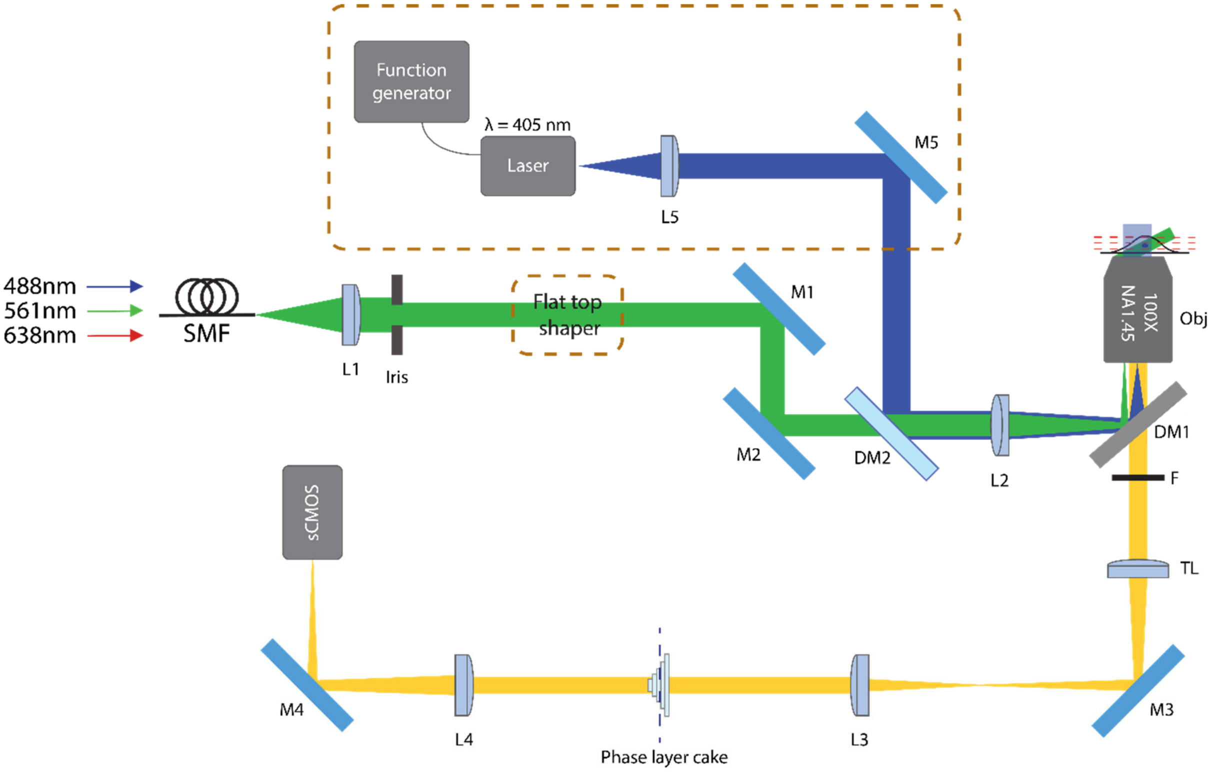

Figure S1. Schematic of 2.5D microscope. L1-4, lenses; M1-5, mirrors; DM1-2, dichroic mirrors; F, filter; TL, tube lens; SMF, single mode fiber; Obj, objective lens. M2 was used to generate the HILO illumination. For STORM imaging, a flat top shaper and an activation laser (405 nm) were inserted. A function generator was used to modulate the illumination of 405nm laser. The layer cake was placed at the conjugate back focal plane of the objective lens.

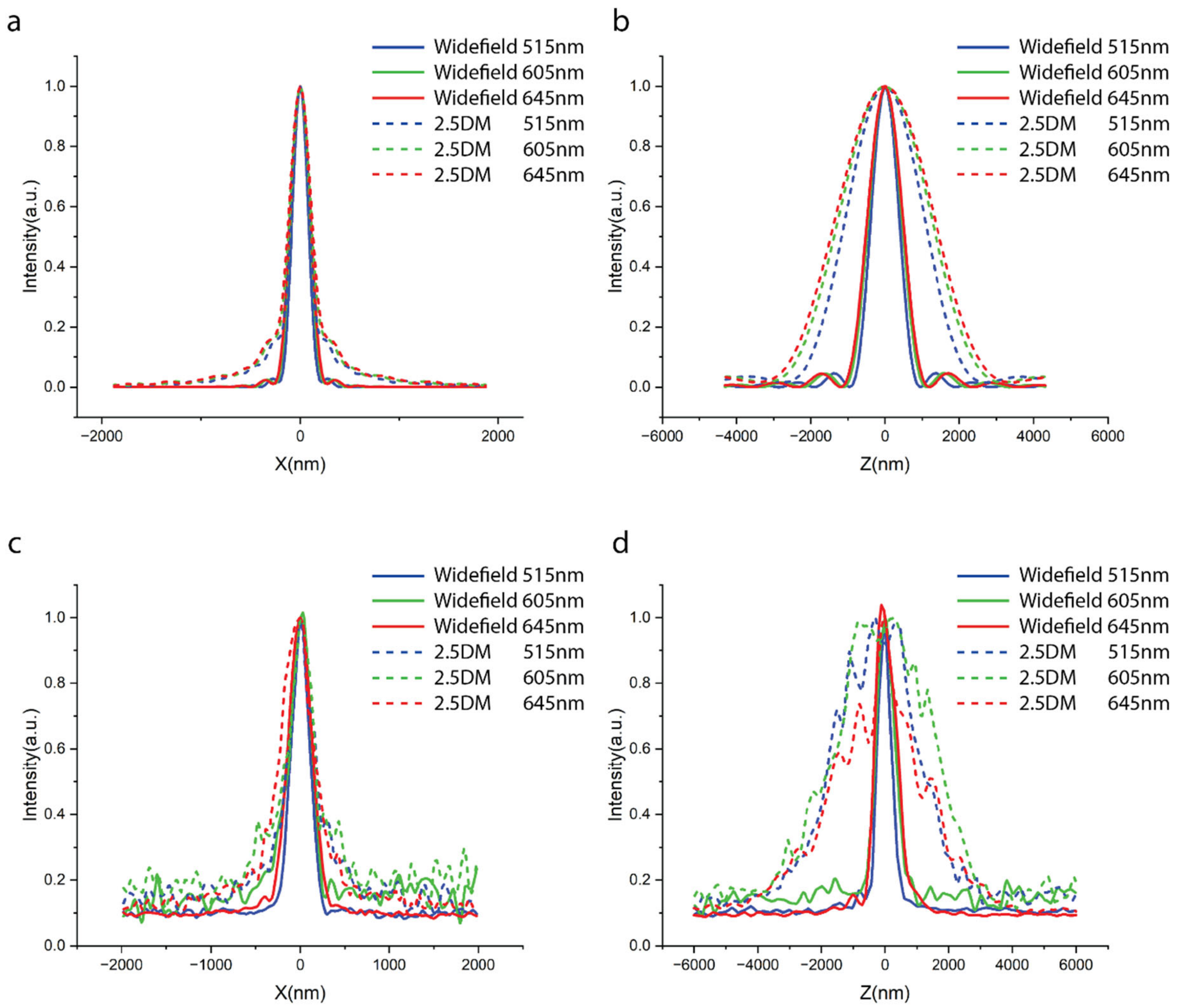

Figure S2. Comparison of the line profiles of PSFs for widefield and 2.5D microscopy. Simulated point spread function line profiles of three colors along the (a) x-axis and (b) z-axis. Experimentally measured line profiles using 100 nm beads along the (c) x-axis and (d) z-axis.

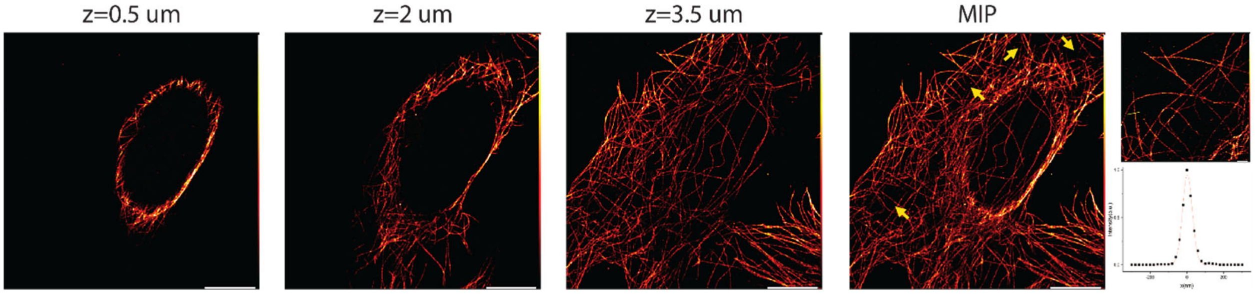

Figure S3. SMLM immunofluorescence images of microtubules labeled with AF647 in U2OS cells captured at different depths using widefield microscopy. Note that some structures are discontinuous after MIP of three depths (pointed with yellow arrows). Scale bar, 10 µm.

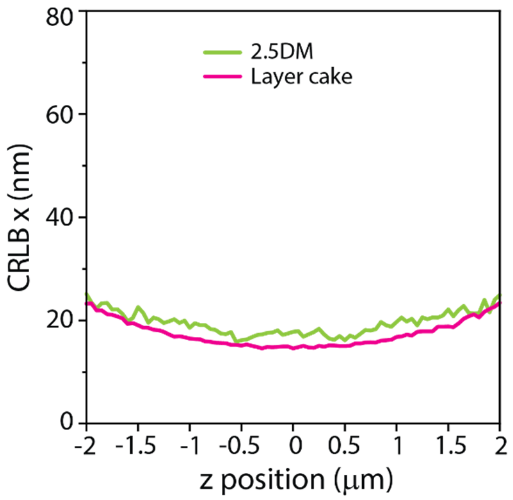

Figure S4. Cramér-Rao lower bound (CRLB) calculations for estimating location (x) of single-molecules with SLM-based 2.5DM (green) and layer cake (magenta) for a 4 µm z range.
